# How much can we save by applying artificial intelligence in evidence synthesis? Results from a pragmatic review to quantify workload efficiencies and cost savings

**DOI:** 10.3389/fphar.2025.1454245

**Published:** 2025-01-31

**Authors:** Seye Abogunrin, Jeffrey M. Muir, Clarissa Zerbini, Grammati Sarri

**Affiliations:** ^1^ Roche, Basel, Switzerland; ^2^ Cytel, Inc., Toronto, ON, Canada; ^3^ Cytel, Inc., London, United Kingdom

**Keywords:** artificial intelligence, systematic review, evidence synthesis, efficiencies, machine learning

## Abstract

**Introduction:**

Researchers are increasingly exploring the use of artificial intelligence (AI) tools in evidence synthesis, a labor-intensive, time-consuming, and costly effort. This review explored and quantified the potential efficiency benefits of using automated tools as part of core evidence synthesis activities compared with human-led methods.

**Methods:**

We searched the MEDLINE and Embase databases for English-language articles published between 2012 and 14 November 2023, and hand-searched the ISPOR presentations database (2020–2023) for articles presenting quantitative results on workload efficiency in systematic literature reviews (SLR) when AI automation tools were utilized. Data on efficiencies (time- and cost-related) were collected.

**Results:**

We identified 25 eligible studies: 13 used machine learning, 10 used natural language processing, and once each used a systematic review automation tool and a non-specified AI tool. In 17 studies, a >50% time reduction was observed, with 5-to 6-fold decreases in abstract review time. When the number of abstracts reviewed was examined, decreases of 55%–64% were noted. Studies examining work saved over sampling at 95% recall reported 6- to 10-fold decreases in workload with automation. No studies quantified the economic impact associated with automation, although one study found that there was an overall labor reduction of >75% over manual methods during dual-screen reviews.

**Discussion:**

AI can reduce both workload and create time efficiencies when applied to evidence gathering efforts in SLRs. These improvements can facilitate the implementation of novel approaches in decision making that consider the real-life value of health technologies. Further research should quantify the economic impact of automation in SLRs.

## Introduction

Automation through artificial intelligence (AI) has been considered the most rapidly evolving field in healthcare and related research. AI has the potential to analyze large pools of diverse data and process heterogeneous information following structured prompts/instructions, and its applications to healthcare, from facilitating early diagnosis and monitoring, to improving overall patient access and quality and efficiency of care, have been increasingly documented (Alami et al., 2020). To date, wider AI applications in health economics and outcomes research (HEOR) and research to inform policy-making (including health technology assessment [HTA]) have failed to gain significant traction. The recent AI position statement by the HTA body in England (National Institute for Health and Care Excellence [NICE]), which sets up the principles around the use of AI methods in the generation and reporting of evidence for the technology submissions, may change the HEOR *status quo* and influence other HTA bodies around the world (NICE, 2024).

The application of machine learning (ML) in pharmacoepidemiology and HEOR has been previously used to advance cohort or feature analytics (confounder adjustment, causal inference) and to predict clinical response or adverse reactions to a drug (Padula et al., 2022; Wyss et al., 2022). Recently, and most apparently during and after the COVID-19 pandemic, HTA bodies have recognized significant challenges in how to process a higher volume of evidence efficiently and rigorously paralleled with a demand to consider a wider evidence base and deliver decisions under short notice (Hair et al., 2021; Daniels et al., 2015). This is equally true in systematic literature reviews (SLR), a cornerstone of evidence-based medicine and policy-making in healthcare decision-making, which aims to identify and synthesize data and/or information for a targeted population or disease problem in a reproducible and unbiased manner. SLRs are, however, labor intensive and costly (Michelson and Reuter, 2019), often taking months to complete and requiring significant effort and training from a team of researchers (Bashir et al., 2018; Shojania et al., 2007). A 2017 analysis using data from the PROSPERO registry confirmed that the time and staff needed to conduct systematic reviews was considerable (Borah et al., 2017), with reviews routinely requiring 6 months and, in more complex topics, several years for completion (Featherstone et al., 2015; Ganann et al., 2010; Khangura et al., 2012). In a 2018 case study, for example, the average time to complete a systematic review was 66 weeks and 881 person-hours (Pham et al., 2018). However, given the increased demands by policymakers to explore more complex methodologies to increase trust in data and provide sound evidence for their decision-makers (such as bias quantification methods, surrogate analyses, and long-term survival extrapolations), it remains a struggle for all stakeholders involved (decision-makers, pharmaceutical industries, researchers) of how to prioritize personnel training and resource needs to meet higher evidentiary needs and stricter methodological requirements while ensuring the evidence produced is up-to-date and findings are timely, relevant, and accurate for decision-making (Sarri et al., 2023). Therefore, the concept of living (regularly updating) systematic reviews (LSR) was introduced as a novel approach to evidence identification and synthesis that aims to continually update a review, using rigorous methodology, to incorporate relevant new evidence as it becomes available (Community, 2024).

In response to these challenges, researchers have begun to embrace AI tools that show how to increase efficiency in SLRs through automation and active learning. In addition to ML, text mining, natural language processing (NLP) and deep learning are all layers that can be grouped under the broad and ever-evolving umbrella of AI and offer a potential solution to the challenges faced by today’s evidence synthesis researchers (Hirschberg and Manning, 2015; Singh et al., 2023). In essence, these tools aim to complete specific review tasks through different applications (active learning, human or researcher in the loop learning) (van de Schoot et al., 2021) by incorporating probabilistic reasoning to deal with uncertainty in the decisions and whereby the algorithms improve with data experience. ML now underpins most of modern AI and can be unsupervised, seeking a pattern in the data presented to it, or supervised, when it learns from information fed into it by a human who has labeled it (for example, by adding the definitive excluded code to screening) (Table 1). In evidence synthesis, AI automation technologies are largely suggested as a method to assist in the time-consuming screening of citations, as this represents the rate-limiting step in the timely completion of SLRs and HTAs (Beller et al., 2013), although previous SLRs have also covered automation processes for all SLR steps (search strategies, text mining, data extraction, synthesis) (Jonnalagadda et al., 2015; O'Mara-Eves et al., 2015; Marshall and Wallace, 2019). A recent review of economic models submitted to the National Institute for Health and Care Excellence outlined shortcomings, both structural and methodological, of the current approaches to literature reviews and highlighted the opportunities available with the expanded use of AI (Daly et al., 2022).

**TABLE 1:** AI definitions related to automation in evidence synthesis.

Types of artificial intelligence	Definition	Focus	Relationship to AI
Machine learning	Computer algorithms which “learn” to perform a specific task through statistical modeling of (typically large amounts of) data	Learning from data to improve performance	Foundational technology for most AI applications
Natural language processing	Computational methods for automatically processing and analyzing “natural” (i.e., human) language texts	Meaning and intent behind text	Core subfield of AI
Text classification	Automated categorization of documents into groups of interest		
Text mining	Process of extracting information and patterns from unstructured text data	Quantitative insights and patterns	No strictly AI but often used in AI applications
Classifiers	Algorithms that learn to assign data points so specific categories	Classification of data	Fundamental concept in AI and ML
Algorithms	Set of instructions for solving a problem	Specific steps to achieve a goal	Foundational building blocks of AI and ML
Data extraction	The task of identifying key bits of structured information from texts		
Crowd-sourcing	Decomposing work into micro-tasks to be performed by distributed workers		
Support vector machines	Machine learning algorithm used for classification and regression tasks	Finding hyperplanes that best separate data points	Subfield of ML used with in AI applications
Micro-tasks	Discrete units of work that together complete a larger undertaking		
Semi-automation	Using machine learning to expedite tasks, rather than complete them		
Human-in-the-loop	Workflows in which humans remain involved, rather than being replaced		
Supervised learning	Estimating model parameters using manually labelled data		
Distantly supervised	Learning from pseudo, noisy “labels” derived automatically by applying rules to existing databases or other structured data		
Unsupervised	Learning without any labels (e.g., clustering data)		
Deep learning	Subfield of ML using artificial neural networks with multiple layers	Learning complex representations from data	Specialized form of ML used within AI
Generative AI	Subfield of AI focused on creating new data, often similar to existing data	Generating new data based on learned patterns	Can be used within other AI subfields like natural language processing

Abbreviations: AI, artificial intelligence; ML, machine learning.

Previous reports have detailed the types of automated technologies available for each stage of evidence synthesis and their impact on results including proposed frameworks for their implementation and barriers to their adoption (Padula et al., 2022; Marshall and Wallace, 2019; Hocking et al., 2022). However, much less effort has been placed on quantifying their impact in terms of efficiencies and cost savings through a review of existing literature.

Against this background, we conducted a pragmatic literature review to explore and quantify the potential benefits gained from the introduction of AI-automated tools in core evidence literature review steps (screening, data extraction, reporting). Our aim was to comprehensively examine the literature to determine whether the use of AI-automated tools was associated with time efficiencies and/or cost savings as compared with traditional (manual) human-led methods. Issues around the development and training of AI tools including metrics around their accuracy and reproducibility of results, although crucial in generating trust for their broader applications and adoption in literature reviews, were topics beyond the scope of this review.

## Materials and methods

Following a pre-designed review protocol, we conducted a structured search of the Embase and MEDLINE databases for English-language, publicly available literature published between 2012 and 14 November 2023 using key AI-related terms: “artificial intelligence”, “machine learning”, “automation”, “web application”, and “living”, the latter of which we included to reflect the regular updating of evidence in SLRs and HTAs that is often accomplished by leveraging automation. A supplementary hand search was conducted of The Professional Society for Health Economics and Outcomes Research (ISPOR) presentations database for related abstracts between 2020 and 2023. Reporting was guided by the Preferred Reporting Items for Systematic Review and Meta-analysis (PRISMA) guidelines (Page et al., 2021). We searched for and included published, freely available articles presenting quantitative results on workload efficiency estimates such as reduced time to undertake any tasks related to performing systematic reviews using a type of automation compared with human effort and/or corresponding cost savings. For the purposes of this review, we considered automated tools to be any instrument or system fitting under the general AI umbrella. A good introduction of systematic review automation is provided by Marshall and Wallace (2019), and readers can familiarize themselves with the terminology of AI and ML tools in research synthesis as listed in Table 1.

Studies referring to the role of AI as part of HEOR-related methods such as databases/patient cohort identification, causal inference, predictive modelling or economic analyses were excluded. We also excluded studies presenting methodological guidelines on AI use and commentaries on this topic. Screening of titles/abstracts and full texts was carried out independently by two (human) reviewers with any disagreements resolved by a third, more senior reviewer.

Data extraction was completed by a single reviewer using a pre-specified template and validated by a second reviewer. We extracted key publication and AI characteristics but also included key themes and comments regarding the key outcomes of interest (decreased workload, time and cost savings). We sought articles that evaluated any efficiencies by using metrics including time-to-review (i.e., for abstracts, *etc.*), number-to-review (i.e., number of abstracts reviewed) or work saved over sampling at 95% recall (WSS@95%). WSS@95% measures efficiency by providing an estimate of the work saved while screening for eligible articles, when compared with traditional manual screening, to find 95% of eligible articles (Cohen et al., 2006; Chai et al., 2021). Data outlining quantified savings in time, cost and/or workload were extracted as reported in the source literature. Data is presented narratively with no pooling of data. Performance metrics of individual tools including specific considerations in their development and validation were beyond the scope of this paper and were not extracted or presented subsequently.

## Results

**FIGURE 1:**
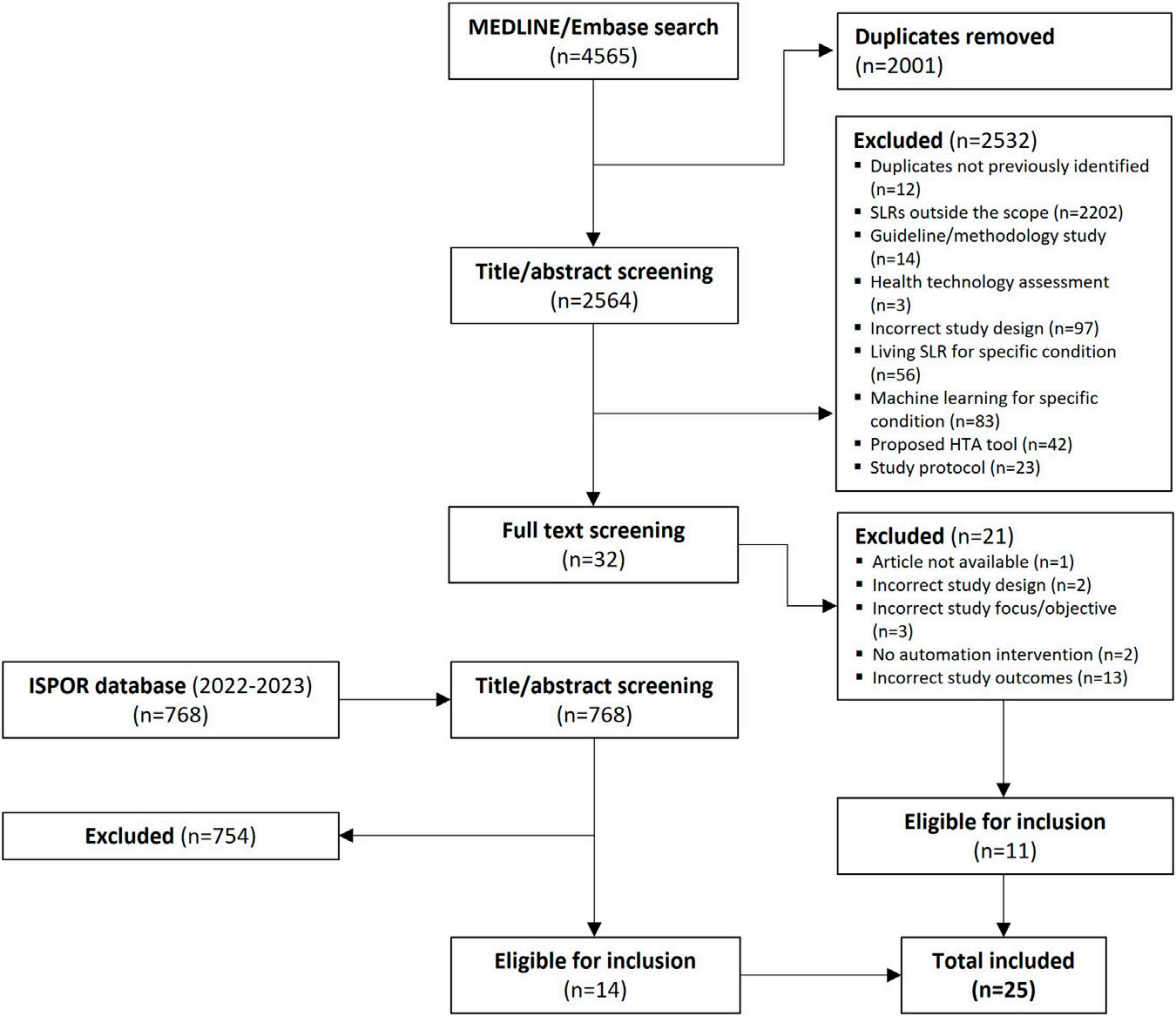
PRISMA diagram summarizing study eligibility and screening process.

In total, 2,564 studies were eligible for title/abstract review after de-duplication, of which 32 proceeded to full-text screening. Eleven studies were deemed to be eligible for inclusion based on full-text review. An additional 14 studies were included via a hand search of the ISPOR presentations database, resulting in a total of 25 studies (Pham et al., 2018; Borowiack et al., 2023; Cichewicz et al., 2023; Egunsola et al., 2023; Liu et al., 2023a; Liu et al., 2023b; Venkata et al., 2023; Bhagat et al., 2022; Hubscher et al., 2022; Liu et al., 2022; Rajadhyax et al., 2022; Stansfield et al., 2022; Clark et al., 2021; Gates et al., 2021; Queiros et al., 2020; Queiros et al., 2022; van Haastrecht et al., 2021; Abogunrin et al., 2020; Popoff et al., 2020; Kebede et al., 2023; Qin et al., 2021; Witzmann et al., 2021; Yamada et al., 2020; Ji and Yen, 2015; Jonnalagadda and Petitti, 2013) that were eligible for inclusion in this review. The literature attrition is shown as a PRISMA diagram in Figure 1.

### Included publications

All 25 studies (Pham et al., 2018; Borowiack et al., 2023; Cichewicz et al., 2023; Egunsola et al., 2023; Liu et al., 2023a; Liu et al., 2023b; Venkata et al., 2023; Bhagat et al., 2022; Hubscher et al., 2022; Liu et al., 2022; Rajadhyax et al., 2022; Stansfield et al., 2022; Clark et al., 2021; Gates et al., 2021; Queiros et al., 2020; Queiros et al., 2022; van Haastrecht et al., 2021; Abogunrin et al., 2020; Popoff et al., 2020; Kebede et al., 2023; Qin et al., 2021; Witzmann et al., 2021; Yamada et al., 2020; Ji and Yen, 2015; Jonnalagadda and Petitti, 2013) examined improvements in time efficiency associated with automation in screening, updating and/or analysis during the SLR process, whereas one study (Cichewicz et al., 2023) examined the impact on labour reduction from the inclusion of automation in this process (Table 2). No studies were identified to quantify the economic (cost savings) impact linked to automation in evidence synthesis.

**TABLE 2:** Eligible studies.

References	Innovation and short description	Therapeutic area	Evidence base	Evidence synthesis task	Innovation role	Time savings (%)	Time savings	Costs savings
Time-to-review								
Borowiack et al. (2023)	Machine learning^a^ Duplication of published SR-CCEO	Osteoarthritis	4,459 records/economic SLR	Abstract screening	Second reviewer	42.69%	19 h	NR
Cichewicz et al. (2023)	Machine learning DistillerAI compared with human reviewer(s)	NR	∼5,000 hits (epidemiology, SLRs/NMAs, treatment guidelines and patterns, utilities)	Abstract screening	Second reviewer	Average: 40% (36%–45%)	NR	39.8%
					Reviewer replacement	Average: 80% (73%–91%)	NR	79.6%
Egunsola et al. (2023)	Natural language processing LiveNMA and LiveSLR used to update NMA/SLR	Prostate cancer	Replication of published NMA	Replicate network and treatment hierarchy	Automated literature updates and results synthesis	NR	NR (time taken for NMA replication: 2 min)	NR
Liu et al. (2023a)	Natural language processing LiveSLR platform used as part of ongoing updates of SLRs	NR	1,595 references	Data extraction	Limited data extraction for GVD updates	99.6%	264.5 h	NR
Liu et al. (2023b)	Natural language processing LiveSTART platform used as part of ongoing updates of SLRs	NR	1,400 hits/24 oncology and non-oncology indications (clinical, economic, humanistic burden SLRs)	Abstract screening	Adaptation to protocol changes	75%	3 h	NR
Venkata et al. (2023)	Natural language processing DistillerAI and Classifiers used to complete 8 TLRs and 3 SLRs	NR	∼5,000 records	Title and abstract screening	Reviewer support system	57.3% (SLRs) – 77.9% (TLRs)	263 h (SLRs) – 311 h (TLRs)	NR
Bhagat et al. (2022)	Natural language processing AI classifier compared with human reviewer when determining eligibility	NR	574 records/no information	Abstract screening	Second reviewer	50%–60%	14–17 h	NR
Hubscher et al. (2022)	Machine learning Ongoing evidence updating using LiveRef	Multiple myeloma	188 hits/targeted reviews in oncology	All steps^b^	Automated literature updates and results synthesis	63%	5 weeks	NR
Liu et al. (2022)	Natural language processing LiveNMA and LiveSLR used to update NMA/SLR	Multiple myeloma	Replication of ICER-produced NMA	All steps	Automated literature updates and results synthesis	NR	NR (time taken for NMA replication: 10 min)	NR
Rajadhyax et al. (2022)	Machine learning AI tool reviewed abstracts for targeted literature review	NR	487 records	Title and abstract screening	Reviewer replacement	40%–50%	3.3 h^c^	NR
Stansfield et al. (2022)	Machine learning AI classifier compared with manual screening	NR	5,812, 7,185 and 9,368 records in 3 test sets	Abstract screening	Reviewer support system	41%–74%	25 h	NR
Clark et al. (2021)	Systematic review automation tools (n = 5) SRA tool used in screening in SLR compared with human reviewers	Chronic kidney disease	596 titles/abstracts as part of a systematic review	Abstract screening	Reviewer replacement	71.6% total 70.3% (screening) 78.6% (learning)	30 h (screening) 5 h (learning)	NR
Gates et al. (2021)	Machine learning and text mining tool AI identification of data elements and relevance in RCTs	NR	Reviewing included 75 RCTs	Abstract screening	Reviewer support system	17.1%	3.7 h	NR
					Reviewer replacement	44.6%	24.4 h	NR
Pham et al. (2018) ^d^	Machine learning and text mining tool Abstract eligibility prediction based on title/abstract training	NR	Published SLR (14,314 abstracts) and published scoping review (17,200 abstracts)	Abstract screening	Second reviewer replacement	55%–63%	91–95 h	NR
Queiros et al. (2022)	Support vector machines Review of human-performed retrospective SLRs	NR	∼44,000 records across different indications/study designs	Abstract screening	Second reviewer	NR	Up to 283 h^e^	NR
van Haastrecht et al. (2021)	Machine learning (SYMBALS) Backwards snowballing combined with machine learning	NR	2,708 papers	Title/abstract and full-text screening	Reviewer replacement	Title/abstract review time was thus improved by a factor of 6	37.5 h	NR
Abogunrin et al. (2020)	Machine learning Comparison of human abstract review with review by two MLMs	Prostate cancer	2,434 records/clinical SLR	Abstract screening	Second reviewer	70%	60 h	NR
Popoff et al. (2020)	Text mining and machine learning Abstract eligibility determination of previously published SLRs	Psoriasis, lung cancer, liver cancer, melanoma, obesity	Five datasets from various disease areas (psoriasis, lung cancer, liver cancer, melanoma and obesity) totaling 33,994 abstracts	Abstract and full-text screening	Reviewer replacement	NR	7.5 h	NR
Queiros et al. (2020)	Support vector machines AAMs review abstract records from human-conducted SLR	Non-small cell lung cancer	5,820 records	Abstract screening	Reviewer replacement	72%	144 h	NR
Number-to-review								
Kebede et al. (2023)	Text-mining and machine learning Validation of eligibility determination using multiple methodologies	Obesity	9,857 records	Abstract screening	Reviewer support system Reviewer replacement	61%–80.3%	NR	NR
Pham et al. (2018)	Text-mining and machine learning Abstract eligibility prediction based on title/abstract training	NR	Published SLR (14,314 abstracts) and published scoping review (17,200 abstracts)	Abstract screening	Second reviewer replacement	55%–63%	91–95 h	NR
Qin et al. (2021)	Natural language processing (LightGBM) Title/abstract eligibility verification of published SLR	Diabetes mellitus	Set of 947 citations	Abstract screening	Reviewer replacement	64.1%	NR	NR
WSS@95%								
Witzmann et al. (2021)	Text mining Classification of title and abstract for inclusion in SLR	Oncology	5 review topics (clinical, economic, utility)/records range from 288 to 9,123	Title and abstract screening	Reviewer support system	WSS@95 ≥ 61%	NR	NR
Yamada et al. (2020)	Machine learning Confirmation of eligibility using SLR and MA of published clinical guidelines	Diabetes mellitus, cardiovascular disease	8 published SLRs	Abstract screening	Reviewer replacement	6-fold (and up to 10-fold)	90%	NR
Ji and Yen (2015)	Text mining Predictive performance of MEDLINE elements	NR	15 published SLRs	Title/abstract screening including Medline elements	Reviewer support system	>36%	NR	NR
Jonnalagadda and Petitti (2013)	Text mining Relevance feedback algorithm	Multiple, drug-related	15 published SLRs	Abstract screening	Reviewer support system	Median 13% (6%–30%)	NR	NR

^a^ This study used a three-stage screening process by training both humans and AI model.

^b^ Tested for rapid updates on topics (epidemiology, disease burden, treatment practices, comparative effectiveness) to support global value dossiers.

^c^ However, humans required extra two hours for cross-checking exclusions and take decisions for unclear references.

^d^ Pham et al. study evaluated both time-to-review and number of abstracts to review.

^e^ Depending on disease area and type of evidence reviewed: range from 5.1 h savings (early NSCLC/trial SLR) to 282.5 h (metastatic NSCLC SLR) using the binary classifier method to range from 0.8 h (early NSCLC/trial) to 276 h (metastatic NSCLC SLR) using the ensemble classifier.

Abbreviations: AAM, advanced analytic methods; GVD, global value dossier; LightGBM, light-gradient boosting machine; MA, meta-analysis; MLM, machine learning methods; NMA, network meta-analysis; NR, not reported; RCT, randomized controlled trial; SLR, systematic literature review; SR-CCEO, systematic review with costs and cost-effectiveness outcomes; SYMBALS, SYstematic review Methodology Blending Active Learning and Snowballing; TLR, targeted literature review; WSS@95%, work saved over sampling at 95% recall.

### Nature of automation

Thirteen (Pham et al., 2018; Borowiack et al., 2023; Hubscher et al., 2022; Stansfield et al., 2022; Gates et al., 2021; Queiros et al., 2020; Queiros et al., 2022; van Haastrecht et al., 2021; Abogunrin et al., 2020; Popoff et al., 2020; Kebede et al., 2023; Witzmann et al., 2021; Yamada et al., 2020) studies used nine different tools to implement 15 different AI methods. Eleven methods (73%) were used in the screening stage of the review. The remainder were divided as follows: two methods (13%) focused on data extraction and two (13%) focused on risk-of-bias assessment. The ambiguous benefits of the data extractions, combined with the reported advantages, indicate that AI platforms have taken hold with varying success in evidence synthesis. However, the results are qualified by the reliance on the self-reporting of study authors.

Regarding specific methods used, 10 studies (Cichewicz et al., 2023; Egunsola et al., 2023; Liu et al., 2023a; Liu et al., 2023b; Venkata et al., 2023; Bhagat et al., 2022; Liu et al., 2022; Qin et al., 2021; Ji and Yen, 2015; Jonnalagadda and Petitti, 2013) used NLP, one study (Clark et al., 2021) used a systematic review automation (SRA) tool and one study (Rajadhyax et al., 2022) used a general AI tool (i.e., not specifically defined). While the application of ML techniques varied somewhat among studies, all those that used ML utilized some variation on the traditional ML approach where results from human searches are used to “train” the ML algorithm, after which potentially eligible citations were classified and categorized by the automation tool. Studies that utilized NLP were generally used to provide updates to existing databases and/or reviews and relied upon NLP as part of the screening process.

### Effect of automation on workload and time saving

Improvements in time efficiency were evaluated in three ways: time-to-review (19 studies) (Pham et al., 2018; Borowiack et al., 2023; Cichewicz et al., 2023; Egunsola et al., 2023; Liu et al., 2023a; Liu et al., 2023b; Venkata et al., 2023; Bhagat et al., 2022; Hubscher et al., 2022; Liu et al., 2022; Rajadhyax et al., 2022; Stansfield et al., 2022; Clark et al., 2021; Gates et al., 2021; Queiros et al., 2020; Queiros et al., 2022; van Haastrecht et al., 2021; Abogunrin et al., 2020; Popoff et al., 2020), number of abstracts screened (three studies) (Pham et al., 2018; Kebede et al., 2023; Qin et al., 2021) and WSS@95% (four studies) (Witzmann et al., 2021; Yamada et al., 2020; Ji and Yen, 2015; Jonnalagadda and Petitti, 2013); one study (Pham et al., 2018) used both time-to-review and number of abstracts (Table 1). All 19 studies using time-to-review as their primary outcome observed substantial time savings with the use of AI. Among these, 15 studies reported on the total time required to review abstracts, with the improvement in time-to-review ranging from 36.0% (Cichewicz et al., 2023) to more than 99.0% (Liu et al., 2023a). Of the full 25 included studies, 17 found a >50% time reduction. The largest time savings noted among the eligible studies was associated with the use of an AI tool designed to provide live updates to SLRs (Liu et al., 2023a). Several studies of this “live update” technology have examined its efficiency and found that in two studies (Liu et al., 2023a; Liu et al., 2023b) time savings ranged from 75.0% to 99.8% while in two other studies (Egunsola et al., 2023; Liu et al., 2022) estimated the time to replicate network meta-analyses (NMA), time savings ranged from two to 10 min to complete the entire analysis, representing time savings of 99.0% compared with a fully manual process completed by human reviewers. The time required per task was drastically decreased with AI automation, with one study (Stansfield et al., 2022) reporting that the time to review individual abstracts could be as low as 7 seconds (compared with approximately 60 s per record by humans (Devane et al., 2014)), which itself contributed to an estimated savings of 25 h in that study. Another study (Egunsola et al., 2023) reported a total time of two minutes to replicate a full NMA. Clark et al. (2021) compared the time-to-review in two teams (one manual, one automated) reviewing the eligibility criteria for a single published systematic review and noted a 72.0% decrease in time required, with manual reviewers requiring 41 h and 33 min to complete the eligibility screening, as compared with only 11 h and 48 min for reviewers assisted by automation.

Another study (Cichewicz et al., 2023) indirectly estimated cost savings based on the hours required to complete tasks estimating a labor reduction of more than 75.0% with associated cost savings of 79.6% (range: 73.0%–91.0%) during dual-screen reviews (i.e., where AI acts as a single screener). For single-screen reviews (i.e., where AI was employed as a second reviewer), they estimated a decrease in hours-to-complete of 33.0%, which translates to costs savings averaging 39.8% (range: 36.0%–45.0%) based on the anticipated manpower saved.

Several studies used a defined number of abstracts for review and compared the time required for manual versus automated/assisted methods. Again, significant efficiencies were observed. Two studies, one in which more than 2,700 abstracts were screened (van Haastrecht et al., 2021) and the other that involved screening of over 33,000 abstracts (Popoff et al., 2020), noted five-to six-fold decreases in the time required to review the abstracts, findings that were observed to be scalable and consistent. Another study (Gates et al., 2021) observed that, when reviewing 75 abstracts, automation used to assist reviewers resulted in a 17.1% time savings, but that when automation was used to replace reviewers, up to 44.6% of time was saved. Elsewhere, ML algorithms were able to complete in 31 min what would have taken an individual reviewer up to 85 h to complete (Popoff et al., 2020), and an estimated total of 25 h of screening time was saved when three sets of citations totaling more than 22,000 citations were screened using AI tools (Stansfield et al., 2022).

Three studies (Pham et al., 2018; Kebede et al., 2023; Qin et al., 2021) evaluated the impact of automation on the number of abstracts reviewed, all of which noted similar decreases in the screening burden of between 55.0% and 64.1%. All three studies compared automated review with manual review and concluded that automation (i.e., ML) has the potential to replace at least one reviewer in the screening process. One study (Kebede et al., 2023) calculated that the minimum decrease in review burden was 61.0% (based on a total of more than 9,800 potential abstracts for review) but that if ML was used to replace one of two reviewers, the workload reduction could be up to 80.3%. All studies reported high sensitivity as well, indicating that quality is not lost when automation is used to replace one of the reviewers. One study (Pham et al., 2018) that used both time to review and number of abstracts reviewed observed that the use of ML not only decreased the number of abstracts reviewed by up to 63.0% but also calculated that this translated to a savings of between 91 and 95 h per systematic review, suggesting that the impact of automation can significantly improve the efficiency of the SLR screening process.

Finally, four studies (Witzmann et al., 2021; Yamada et al., 2020; Ji and Yen, 2015; Jonnalagadda and Petitti, 2013) used WSS@95% to quantify the reduction in workload associated with automation, reporting between six- and 10-fold improvements in workload with ML. In one study (Yamada et al., 2020), ML was compared with manual screening for eight previously published systematic reviews. The authors reviewed published SLRs and extracted both correct (those reviewed as part of the published SLR) and incorrect (those not reviewed as part of the published SLR) articles and used those articles to train an AI algorithm. They noted that a six-fold decrease in workload was observed when all articles were used but that when two correct articles were randomly selected by a researcher and used to initiate ML, the process was accelerated further, with a maximum 10-fold decrease in workload observed. Elsewhere, two studies used methods relating to text word searching and found that efficiencies improved by up to one-third. One of them (Ji and Yen, 2015) used MEDLINE elements such as TI (title), AB (abstract), MH (MeSH heading), PT (publication type) and AU (author name) in various combinations and noted average improvements of between 36.0% and 37.0%, while the other study (Jonnalagadda and Petitti, 2013) used distributional semantics to assist with abstract text word screening and noted improvements ranging from 6.0% to 30.0% (median: 13.0%). Finally, in one study (Witzmann et al., 2021) that used text mining to evaluate five review topics (three clinical, one economic, and one utility review) with datasets varying from 288 to 9,123 articles, there was a >61.0% reduction in the number of articles needing manual review for all topics.

## Discussion

AI technologies and methods to speed up the production of SLRs by reducing manual burden and promote cost savings have recently emerged. To date, much of the discussion has focused on the type of each AI tool and its validation properties and computing technicalities, with less emphasis on systematically generating evidence around these tools’ efficiency metrics in SLRs. To close this gap, our pragmatic review sought to quantify the impact of using automated tools in evidence synthesis (review, economic modelling) in terms of efficiencies and cost savings and provide an evidence base for the implementation of automation in SLR methodologies.

The innovation of SLR automation tools (e.g., ML algorithms, automated screening, and automated data extraction) and advanced web-based economic models using cloud-based tools can have a significant impact on time and given a presumed decrease in manpower, potential cost savings for both manufacturers and decision makers. van Dijk et al. (2023) have shown that the use of an AI reviewer can reduce the number of articles to be reviewed by human reviewers to as low as 23.0%, which aligns with other work by Yao et al. (2024) that showed in a systematic review that the time savings using an AI reviewer can range from seven to 86 h when reviewing titles and abstracts. Despite these demonstrated benefits, the uptake of these tools has been slow, mainly due to human factor-related barriers (Tachkov et al., 2022), limited validation of SRA tools (O'Connor et al., 2018) or data-related barriers and distrust of the tools (van Altena et al., 2019) due to lacking transparency of ML systems. Results from this review demonstrated that automation in SLR can substantially decrease the time required to complete the review, decreasing the number of articles required to be reviewed at the full-text level by a factor of five to six and resulting in up to a 10-fold decrease in workload. Our review revealed a literature (data) gap on quantifying the potential economic impact (cost savings) associated with the improved efficiencies through automation in evidence synthesis tasks. We only found one study (Cichewicz et al., 2023) that estimated potential cost savings based on manpower decreases that could indirectly translates to cost savings. Given that human reviewers are known to average 60 s per citation reviewed (Wang et al., 2020), as compared with AI tools that can replicate an entire review in minutes (Liu et al., 2023a; Liu et al., 2023b), significant potential lies ahead with the use of such technologies for conducting SLRs. Michelson and Reuter (2019) recently examined the costs associated with SLR work in the pharmaceutical industry and found that, in the United States, a typical SLR costs in excess of $141,000 when costs for time and manpower are considered. Further, among the 10 largest pharmaceutical companies and 10 largest academic centers, between $16 million and $18 million is spent annually on SLRs (figures which may be underestimated, as it is unclear if they include other types of literature reviews such as pragmatic reviews). Based on our findings that automation can at minimum assist and at best replace one of two reviewers during the identification and screening process, there is the potential for significant savings associated with the decreased labor requirements. More work is needed to accurately quantify these potential savings opportunities, though, as automation itself will be associated with development and implementation costs and, as with any technology, some experience and training in its use (by reviewers) are required to maximize its efficiency. Furthermore, while these results provide important evidence regarding the efficiencies gained through automation, other aspects such as performance metrics of individual tools, safe data access, new structures of data classification and technological acceptance criteria should be factored into future research in this area.

Creating time efficiencies in the SLR process allows for the redistribution of efforts, with the potential to shift the more labor-intensive activities to automation, freeing up human researchers to consider a wider breadth of questions regarding a new technology (beyond the fundamental safety and efficacy questions) and redirect time to resolve more complex methodological topics in comparative effectiveness research. Reason et al. (2024) suggest that this shifting of effort could be exploited to advantage in the development of health economic models, where automation can be used to rapidly review, adapt, and expand existing models, thus removing the delays associated with human action and allowing for the further development of concepts. This is especially valuable in the case of new technologies, which are often associated with a high degree of uncertainty, complicated clinical scenarios but also represent the possibility for considerable added value for patients. Automation in the HTA/SLR environment could therefore support stakeholders, for example, by allowing for the exploration of non-traditional methods for data collection and real-world evaluations to aid AI-based decisions.

The incorporation of AI tools into literature reviews, however, is not without challenges. As with any technology designed and programmed by humans, the quality of the output is dictated largely by the quality of the input. Many publications have previously outlined concerns around diversity and inclusion in AI system design, development, and deployment and have highlighted how ignoring these issues may exaggerate existing discrimination, health inequalities and algorithmic oppression, leading to AI systems being perceived as untrustworthy and unfair (Shams et al., 2023; Chen et al., 2024).

Many researchers acknowledge a trade-off when using AI or automation tools, where AI may allow a task to be completed more quickly but may not be completed to the same standard as when completed by humans (Control ECfDPa, 2022). Additionally, AI tools such as large language models are not themselves deterministic, meaning that responses will not be identical each time a particular question is asked (Qureshi et al., 2023). As a result, some researchers may feel that the use of AI forces users down a specific path of decision-making while not understanding how the tool made specific decisions (Control ECfDPa, 2022). Whether to use AI in a given study is a fundamental decision facing researchers, as is the appropriate choice of stopping criteria (van Dijk et al., 2023). AI algorithms do not autonomously decide where or how to be applied (van de Schoot et al., 2021), as their application relies upon a combination of human skill and training using large volumes of data, which can be difficult to collect due to the ethical implications (Ali et al., 2023). However, in the case of the usage of AI for conducting SLRs, ethical implications are less of a concern as the data being processed are secondary, publicly available data. Regardless of the robustness of input, the evidence we found shows that AI in SLR applications is currently biased heavily toward one predominant task, namely, determination of study eligibility. Although this is a logical first step in applying AI to evidence synthesis tasks, given its labor-intensive nature, the application of AI to other tasks within the SLR, and ultimately HTA, process demands more attention (de la Torre-López et al., 2023).

The efficiency gains noted in one of the included studies (Egunsola et al., 2023) may have been overestimated by ignoring the time required for data preparation and training of the AI tool itself. Nevertheless, some included studies (Queiros et al., 2020; Abogunrin et al., 2020) with more conservative estimates indicate that using an AI tool saves considerable resources even when all preparation and training time is accounted for. A small number of studies in our review considered the utilization of AI for other SLR steps by replicating entire studies, including data analysis. Although these studies demonstrated significant efficiency improvements, these findings require further validation across several disease areas and SLR topics. de la Torre-López et al. (2023), in a comprehensive review of the state of AI technology in SLRs, identified challenges with the AI applications in more advanced tasks beyond simple “selection-based” tasks such as research question formulation, defining of inclusion/exclusion criteria and reporting SLR results and the need for more active human involvement in AI-assisted SLR efforts. Finally, while researchers may embrace the use of AI tools to assist in their efforts, the technology remains a relative “black box”, where end users do not fully understand the inner workings of the technology (Quinn et al., 2021). As such, a potential lack of trust in the automated results may persist and presents a barrier to widespread acceptance of–and expansion of–the use of AI tools in the HTA space (Tachkov et al., 2022; van Altena et al., 2019). Some work has already been done by Abogunrin et al. (2023) to enable the transparent reporting of data generated by AI tools during the conduct of SLRs. Standardized best practices for AI use in HEOR activities, similar to other areas in healthcare, will provide the framework for methods reporting for both researchers and decision-makers and foster trust and transparency in the results produced by these tools (Author Anyonomus, 2021). The “Vienna Principles” established by the International Collaboration for the Automation of Systematic Reviews have outlined the basic principles for automation across the spectrum of review tasks, continuous improvement and how their integration can adhere to high-quality standards (Kraker et al., 2016). The latest PRISMA guidelines already provide some direction on reporting of the use of AI for conducting SLRs, though there is still gap as to the explainability of the underlying algorithms used in tools with AI embedded in them. Likewise, current quality assessment tools do not adequately address quality assessment in automated SLRs. The NICE AI position statement provides the first HTA-developed principles for guiding the integration of AI tools in evidence generation and reporting and can provide the foundation for HTA cross-collaboration efforts in this area. Of note, our review quantitatively supported the NICE statement regarding the less established value demonstration of AI tools, in terms of efficiencies and cost savings, for data extraction steps compared to the tools used for evidence identification. The risk-tier based system suggested by both the EU AI regulation (Europarl, 2023) and NICE position statement (NICE, 2024) should guide reliable and trustworthy integration of such tools in evidence synthesis activities to support decision-making in healthcare.

## Conclusion

Automation through AI is key to unlocking the potential of real-time, dynamic evidence generation creating substantial efficiencies in evidence generation efforts. While AI tools have made significant headway in supporting these processes, challenges and opportunities lie ahead. Integration between tools to facilitate data synthesis remains a prominent gap. Different review topics may require tailored synthesis methods, therefore interoperability between tools is crucial to ensure a smooth flow of data between SLR stages while the necessity for human oversight can build trust in the automated process. Recent examples of automated analysis and reporting for comparative analyses and health economic modeling using open codes have also been proposed, suggesting a wider application of AI tools is possible to allow a real-time monitoring of new evidence in decision-making.

## Data Availability

The original contributions presented in the study are included in the article/supplementary material, further inquiries can be directed to the corresponding author.
